# The GReat-Child Trial^TM^: A Quasi-Experimental Dietary Intervention among Overweight and Obese Children

**DOI:** 10.3390/nu12102972

**Published:** 2020-09-29

**Authors:** Hui Chin Koo, Bee Koon Poh, Ruzita Abd. Talib

**Affiliations:** 1 Nutritional Sciences Programme & Centre for Community Health Studies (ReaCH), Faculty of Health Sciences, Universiti Kebangsaan Malaysia, Kuala Lumpur 50300, Malaysia; koohc@tarc.edu.my (H.C.K.); pbkoon@ukm.edu.my (B.K.P.); 2 Department of Bioscience, Faculty of Applied Sciences, Tunku Abdul Rahman University College, Kuala Lumpur 53300, Malaysia

**Keywords:** children, childhood obesity, intervention, Malaysia, quality of diet, whole grain

## Abstract

Diet composition is a key determinant of childhood obesity. While whole grains and micronutrients are known to decrease the risk of obesity, there are no interventions originating from Southeast Asia that emphasize whole grain as a strategy to improve overall quality of diet in combating childhood obesity. The GReat-Child Trial aimed to improve whole grain intake and quality of diet among overweight and obese children. It is a quasi-experimental intervention based on Social Cognitive Theory. It has a 12-week intervention and 6-month follow-up, consisting of three components that address environmental, personal, and behavioral factors. The intervention consists of: (1) six 30 min lessons on nutrition, using the Malaysian Food Pyramid to emphasize healthy eating, (2) daily deliveries of wholegrain foods to schools so that children can experience and accept wholegrain foods, and (3) diet counseling to parents to increase availability of wholegrain foods at home. Two primary schools with similar demographics in Kuala Lumpur were assigned as control (CG) and intervention (IG) groups. Inclusion criteria were: (1) children aged 9 to 11 years who were overweight/obese; (2) who did not consume whole grain foods; and (3) who had no serious co-morbidity problems. The entire trial was completed by 63 children (31 IG; 32 CG). Study outcomes were measured at baseline and at two time points post intervention (at the 3rd [T1] and 9th [T2] months). IG demonstrated significantly higher intakes of whole grain (mean difference = 9.94, 95%CI: 7.13, 12.75, *p* < 0.001), fiber (mean difference = 3.07, 95% CI: 1.40, 4.73, *p* = 0.001), calcium (mean difference = 130.27, 95%CI: 74.15, 186.39, *p* < 0.001), thiamin (mean difference = 58.71, 95%CI: 26.15, 91.28, *p* = 0.001), riboflavin (mean difference = 0.84, 95%CI: 0.37, 1.32, *p* = 0.001), niacin (mean difference = 0.35, 95%CI: 1.91, 5.16, *p* < 0.001), and vitamin C (mean difference = 58.71, 95%CI: 26.15, 91.28, *p* = 0.001) compared to CG in T1, after adjusting for covariates. However, T1 results were not sustained in T2 when intervention had been discontinued. The findings indicate that intervention emphasizing whole grains improved overall short-term but not long-term dietary intake among schoolchildren. We hope the present trial will lead to adoption of policies to increase whole grain consumption among Malaysian schoolchildren.

## 1. Introduction

Being overweight and obese used to occur only among adults and the elderly. The dramatic increase of childhood obesity globally shows that health habits in children have changed over time. According to the World Health Organization (WHO), Southeast Asia has seen an upward trend in childhood obesity during the last 10 to 15 years; with Malaysia and Brunei having the highest prevalence of obesity among youths aged five to 19 years [1]. Rates of childhood obesity in Malaysia have risen dramatically, from 11.9% in 2015 [2], to 14.8% in 2019 [3].

Recently, attention has focused on improving diet patterns and lifestyle habits in children as a strategy to manage childhood obesity [4]. Interventions to improve the quality of diet and to modify lifestyle habits are vital in managing childhood obesity [5]. While it has been shown that several lifestyle interventions have been successful in improving the body composition of overweight and obese children [6,7], only a handful have evaluated the overall quality of diet in their trial outcomes [8]. 

Food intake is an essential factor associated with the health status of children. Malnourishment from insufficient food used to be a significant child-health concern but dietary patterns have changed over time. Nowadays, especially in developed countries, nutrient-dense foods have been replaced with energy-dense foods, such as convenience and fast foods [9]. This dietary transition is also occurring in Malaysia [10]. This major health concern has led to a dramatic rise in childhood obesity. While childhood obesity is associated with high energy, high fat, and high sugar diets, many children are also unable to obtain the recommended intakes of fiber and other micronutrients, e.g., calcium and B vitamins, which are crucial for growth [9]. Nonetheless, sociodemographic factors, such as gender, household income, parents’ occupation and educational level, also contribute toward childhood obesity in Malaysia [11].

Childhood is a crucial period for shaping dietary patterns and food consumption behavior. These changes carry over into adulthood and as such also reduce the life-long risks of chronic diseases [12]. As the quality of diet is strongly associated with the cognitive and physical development in children [13], a nutritious diet is of utmost importance during childhood. Modifications of dietary patterns and eating behavior are challenging. However, changing parental feeding practices is a possible strategy to manage childhood obesity and improve the dietary pattern in children [14].

Several dietary guidelines recommend that whole grain should make up at least half of all grain products consumed daily [15,16]. A published study has indicated that whole grain intake may improve nutrient intake and quality of diet by delivering a better profile of dietary carbohydrates [17]. Whole grain also contributes significantly to vitamin, mineral, and phytochemical intakes since these components are naturally higher in bran and germ, which are lost during the refining process [17]. Whole grain consumers from the United Kingdom [18] and Australia [19] have nutrient intakes that are closer to dietary reference values. 

## 2. Methodology

### 2.1. Study Design and Recruitment of Participants

In order to achieve a better understanding of causality, our study emphasizes an experimental trial called the GReat-Child Trial. The protocol of this intervention that describes the development of the intervention [20]; its effects on weight management [8]; and knowledge, attitudes, and practices toward whole grain [21] have been published elsewhere. The acceptability and feasibility of the GReat-Child Trial have also been described and published [21]. This present paper describes the effect of the GReat-Child Trial on whole grain intake and quality of diet among overweight and obese Malaysian schoolchildren. 

The GReat-Child Trial was a 12-week non-blinded quasi-experimental intervention, with a 6-month follow-up. The present trial comprised of an intervention (IG) and a control school (CG) in Kuala Lumpur, Malaysia. Schools with similar socio-demographic backgrounds, e.g., educational background, household income, and religious affiliation, were allocated as IG and CG on a non-randomized basis to ensure that the IG and CG groups were completely independent. As the IG and CG schools were at least 10 km apart, the children from both groups did not have any direct or indirect communication with each other [22]. The inclusion criteria were: (1) Malaysian school children between 9 and 11 years old; (2) who were overweight or obese with body mass index-for-age Z-score (BAZ) > +1SD [23]; (3) who can read and write and understand Malay; and (4) who had at least one parent who was willing to attend diet counseling sessions to help resolve their child’s weight problem. Children were excluded if: (1) they had a serious co-morbidity which required treatment; (2) they were on a gluten-free diet; and (3) they indicated during a 3-day diet recall they had consumed whole grain foods. Children who met the inclusion criteria were recruited using the cluster sampling method. The sample size required for the trial was 25 per group by using Naing’s equation (2009) [24], while taking into account a non-response rate of 50%, the required sample size for each group was increased to 38; hence, the total number of children needed in the trial was 76.

The Research Ethics Committee of Universiti Kebangsaan Malaysia (Code: NN-070-2014) reviewed and approved the study protocol. The Ministry of Education, Malaysia and the Kuala Lumpur Federal Territory Education Department granted permission to carry out data collection. Permission was also given by the principals of the selected schools. Parental written, informed consent was acquired prior to baseline data collection. Verbal assent was also obtained from the children before the study began. Children in the IG underwent the 12-week intervention and six months of follow up. The children in the CG did not receive any intervention. However, a 2 h general healthy eating talk was conducted for the CG after the entire GReat-Child Trial was successfully completed. 

The present trial consists of three components: (1) daily eating of whole grain foods (whole grain biscuits, whole grain bread, and whole grain ready-to-eat cereal) during break time at school for 12 consecutive weeks; (2) six nutrition education classes conducted fortnightly, after regular school hours and before the start of after-school co-curricular activities. Each class lasted 30 min and used the food pyramid guide and visual plate model to emphasize whole grains and a healthy balanced diet; and (3) family involvement where a parent attended an hour of one-to-one diet counselling on maintaining a balanced diet and making available whole grain foods at home. 

Whole grain foods were delivered daily during the school break by a dietitian. During the five-day school week, one standard pack of 30 g whole grain ready-to-eat cereals were provided twice a week; two slices of whole grain bread spread with 1 teaspoon of margarine were provided twice a week; while two sachets of whole grain biscuits, which contained 4 pieces of whole grain biscuits, were provided once a week. The selected whole grain foods were served with a 200 mL pack of full-cream milk to replace the food that the children would normally eat during their break. All the distributed whole grain foods were consumed by the children during their break under the dietitian’s supervision. The food items provided were purchased by the researchers and not provided free by any company. If a child was absent on a particular day, the whole grain food would be distributed the next day and taken home for consumption during the weekend. To make sure the children follow the instructions provided, their parents were reminded via phone calls or SMSs on that particular weekend. The aim of food delivery was to provide the children the opportunity to experience healthy whole grain dietary alternatives, where one serving of whole grain food replaced one serving of refined-grain food. The selected whole grain foods were nutrient-dense foods which did not require complicated preparation.

The classroom nutrition education component employed a simplified “visual plate model” and “Food Pyramid Guide” to provide guidance on how to have and maintain a balanced diet with sufficient whole grain intake. This component aimed to improve knowledge of whole grains and balanced diets, reduce intake of refined carbohydrates and substitute them with high fiber whole grain, and recognize the value of whole grain foods. The topics in the module included such topics as the concept of energy balance, the food pyramid, a visual plate model, the importance of whole grains, sources and sampling of whole grain foods, and understanding food labels. At the end of each class, the children used their newly learnt whole grain and balanced diet knowledge to play games. Those who answered questions correctly during the game sessions were presented with stationery sets. The children also received written educational materials that contained the plate model, a dietary guide, and whole grain recommendations discussed earlier in the nutrition education classes.

The family involvement component included a session of individual diet counseling with the parent prior to the 12-week intervention. During this session, the parent was informed individually about their child’s body fat percentage, body mass index-for-age Z-score (BAZ), and waist circumference. These were planned to increase the parental awareness of childhood obesity. In addition, all parents were educated on whole grain and healthy balanced diet recommendations. They were taught to identify whole grain foods, read food labels, and reduce portion sizes. Each parent received written educational materials that contained whole grain recipes and handy tips for preparing whole grain foods, which were discussed during the individual diet counseling session.

### 2.2. Whole Grain Intake Outcome

Whole grain intake was assessed on three occasions: at baseline (T0), at the thirteenth week (T1, after 12-week intervention), and at the ninth month (T2, after 6-month follow-up). Results from the IG and CG were compared. According to the American Association of Cereal Chemists International (AACCI), whole grain includes cereal grains that consist of cracked, ground, or intact grains, which incorporate all of the components of natural grain, including the bran, endosperm, and germ. The components in whole grain cereals have to be present in the same relative proportion as they exist in the intact grains [25]. The AACCI definition of ‘whole grain’ was used in the GReat-Child Trial. There was no lower limit to the whole grain content of a food item for inclusion in the analysis. Whole grain intake for each child was calculated by identifying each whole grain food consumed over the three days of dietary recall. A whole grain consumer was defined as a child who consumed a whole grain food item on at least one of the three recall days. The amount of whole grain per 100 g in each whole grain food item was estimated by one of three methods: (1) using quantitative ingredient declarations on food package labels, (2) directly contacting the manufacturers to obtain the information, or (3) taking an average of the whole grain content of similar products. Details of all whole grain foods, including the total whole grain content per 100 g and the method of calculation, were recorded in an Excel spreadsheet. For each child, the amount of whole grain consumed was estimated by multiplying the actual weight of each whole grain food item consumed by the estimated percentage of whole grain content. Mean daily intakes over the three days of dietary recall were calculated.

### 2.3. Dietary Intake Outcomes

Dietary intakes were also assessed on the same three occasions as whole grain intake, namely at baseline (T0), at the thirteenth week (T1), and at the ninth month (T2). Results from the IG and CG were compared. To assess dietary intakes, 24 h diet recalls for three days (two weekdays and one weekend day) were conducted. Face-to-face interviews were conducted with each child. Tableware items, pictures, and models of commonly consumed foods were used to improve estimation of portion sizes and weights. Nutrient intakes were determined from the three-day diet recalls using NutritionistPro software (Axxya Systems, Stafford, TX, USA), based principally on the Nutrient Composition of Malaysian Food and food product labels. Mean daily intakes over the three days were calculated.

### 2.4. Cut-off Point for Energy Misreporting in Children

Energy intake was compared with estimated basal metabolic rate (BMR) to exclude children who had under-reported or over-reported their energy intake in their 3 day 24 h dietary recalls. BMR was calculated using standard equations based on sex and age provided by FAO/WHO/UNU (1985) [26]. For children in a non-dieting population, Torun et al. (1996) proposed that a ratio between energy intake and BMR of less than 1.39 and more than 2.24 be considered as under-reporting and over-reporting respectively [27]. These cut-off points were applied in the present trial.

### 2.5. Physical Characteristics

Anthropometric measurements were taken to assess physical characteristics. Body weight and height were measured twice, according to standard procedures. The former was measured with a calibrated Tanita digital scale Model SC-330 (Tanita Co., Tokyo, Japan) to the nearest 0.1 kg and the latter with a SECA Bodymeter 206 (SECA GmbH & Co., Hamburg, Germany) to the nearest 0.1 cm. The weight status of the children was determined based on the World Health Organization (WHO) growth reference for children aged 5–19 years old [23]. BAZ was determined using the WHO AnthroPlus version 1.0.3 (World Health Organisation, Geneva, Switzerland) software. 

### 2.6. Statistical Analyses

Statistical analysis was conducted using Statistical Package for the Social Sciences (SPSS) version 22.0 (IBM SPSS Statistics 2014). Each variable was tested using the Shapiro–Wilk test, as our sample size was less than 100. Continuous data were presented as mean and standard deviation (SD), whereas categorical data were presented as number and percentage. The differences between children from the IG and CG were determined using *t*-test for age and household income (continuous data) and chi-square test for sex and household income (categorical data). The baseline differences between the IG and CG across physical characteristics, whole grain, and dietary intakes were determined using an independent *t*-test. The present study applied a complete case analysis, where missing data in the proposed model were dropped from the analysis. This analysis was more efficient than intention-to-treat analysis for a non-longitudinal quasi-experimental pre–post study, as the intention-to-treat approach often included all randomized subjects in the groups to which they had been randomly assigned, regardless of their adherence to the entry criteria, the treatment they actually received, subsequent withdrawal from treatment, or deviation from the protocol [28]. ANCOVA was performed to determine the effect of the GReat-Child Trial over the entire follow-up period, including the 6 months post-intervention follow-up. Two models were examined including: (1) within-group differences and (2) between-group differences. For within-group differences, the entire data set was split into IG and CG. Repeated measures ANCOVA within group analysis was applied, followed by pairwise comparison with confidence interval adjustment. For between-group differences, adjusted mean using repeated measures ANCOVA between group analyses were applied, followed by pairwise comparison. All the models were adjusted for covariates, including all observed pre-treatment variables with some connection to the outcome, all known risk factors for the outcome, and all direct causes of the treatment or the outcome [29]. Hence, household income and baseline variables were considered as covariates in each model in order to prevent bias. Model assumptions, including normality of the residuals, homogeneity of variance, compound symmetry, and homogeneity of regression, were verified. *p*-values of less than 0.05 for a two-sided test were considered statistically significant.

## 3. Results

### 3.1. Baseline Socio-Demographics, Physical Characteristics, Energy Misreporting, and Dietary Intakes among Children Who Completed the 12-Week Trial and 6-Month Follow Up

The participants flow has been described elsewhere [21]. In brief, a total of 122 children who fulfilled the requirements were recruited for the present trial (58 from the CG; 64 from the IG). Of the 122 children, 101 agreed to participate, resulting in a response rate of 82.7%. However, only 63 children successfully completed the entire GReat-Child Trial. Table 1 showed the baseline socio-demographics, physical characteristics, and dietary intakes overview of the 63 children. The children had a mean age of 10.5 years. A majority of them were from families which earned a household income of RM2300-RM5599. Children in both groups were within the obese category based on their mean BAZ. Energy misreporting was prevalent in 4 (6.3%) of 63 children. After excluding the data from children who under- and over-reported their energy intake, a total of 59 children were included in dietary intake analyses. There were no significant differences demonstrated between the IG and CG in socio-demographics, physical characteristics, and energy-misreporting. No between-group differences were observed for macronutrients and fiber intakes. However, the intake of calcium was significantly higher in the CG. 

### 3.2. The Intervention Effects: Within-Group Differences

Table 2 reveals the 9-month changes in whole grain and nutrient intakes within the IG and CG. Significantly higher whole grain, fiber, thiamin, riboflavin, niacin, and calcium intakes were demonstrated in the IG at T1 compared to T0. However, the same group of children showed significantly lower intakes of whole grain, fiber, folic acid, and calcium at T2 compared to T1. A smaller but nevertheless important change was that the IG demonstrated significantly higher whole grain, fiber, thiamin, niacin, calcium, and iron intakes at T2 compared to T0. 

On the other hand, children from the CG showed significantly higher intake of fiber, thiamin, riboflavin, and niacin at T1 compared to T0. Significantly higher intake of fiber, thiamin, and riboflavin at T2 compared to T0 were also shown in the same group of children.

### 3.3. The Intervention Effects: Between-Group Differences

Table 3 showed the differences between the IG and CG in whole grain and nutrient intakes over nine months. The children from IG showed a significantly higher intake of whole grain, fiber, thiamin, riboflavin, niacin, and calcium throughout the study period compared to children from CG. 

## 4. Discussion

The overwhelming prevalence of childhood obesity highlights the importance of nutrition education programs and interventions for children to attain and maintain a healthy weight. Outcomes from the GReat-Child Trial indicated positive improvement in weight management [8], knowledge, attitudes, and practices toward whole grain [21], as well as whole grain intake and quality of diet among the children participants. Considering that whole grain intake among Malaysian children has been a mere 2.3 g/d [30] in the past few years, these positive findings may have significant impact on the long-term dietary habits among Malaysian children. To our knowledge, this is the first reported quasi-experimental trial designed to increase the whole grain consumption, as well as improve the overall quality of diet in Malaysian children who are overweight and obese. 

There were no significant differences between the IG and CG across physical characteristics, demographic characteristics and energy misreporting. This is consistent with recommendations from a published study indicating that the IG and CG should have comparable baseline data [22]. However, the IG and CG showed significant difference in calcium intake. Hence, the GReat-Child Trial used the baseline results as covariate in the data analysis. The application of ANCOVA has been advocated to evaluate the effectiveness of an intervention effect and to adjust the baseline variables as covariates [31]. In an experimental trial, when the IG and CG showed similar mean baseline values, both the ANCOVA model and the post-intervention score method would provide an unbiased estimate of the actual intervention effect [31]. The baseline-controlled method is a statistically effective method to assess causal factors, since dissimilar covariates can be properly adjusted in order to segregate the factors at work. This method is highly useful to researchers because it can adjust for all the major threats to internal validity, e.g., selection, instrumentation, and maturation [32]. 

The strategy of recurrent exposure to similar tastes, in which children are offered the same foods repeatedly, has been shown to be extremely effective in promoting the intake of unfamiliar food by children [33]. Based on the mere exposure theory, if one-time exposure is sufficient to create a positive attitude towards a stimulus, it follows that recurrent exposure to similar tastes will lead to positive acceptance of unfamiliar foods over time [34], while a previous study showed that children increase their consumption of vegetables after being exposed to the taste of vegetables five times [35,36]. In general, children require between eight to ten exposures to a taste, at regular intervals, before they develop a preference for it [37]. This recurrent exposure strategy was applied in the GReat-Child Trial. Although this strategy has had strong results for improving whole grain consumption in children, it should be noted that the sense of taste is not the only sense that comes into play. Other senses such as, sight, smell, and even touch and hearing, also affect the preference for the food. Thus, to allow for regular exposure to the taste of whole grain foods, the GReat-Child Trial supports the idea of having whole grain foods available at home. During the GReat-Child Trial, the witnessed changes in whole grain foods intake can be attributed to the delivery and supervised consumption of whole grain foods on a daily basis and the nutrition education lessons during the 12-week intervention. 

Children in the IG showed significantly higher fiber, B vitamins, and calcium intakes compared to children in the CG over the nine months. As whole grain foods still retain various kinds of vitamins and minerals [38], consumers of whole grain foods expectedly have significantly higher intakes of such vitamins and minerals as calcium, thiamin, and riboflavin. However, it is difficult to determine the effect of a balanced diet and lifestyle which accompanies whole grain intake in the IG. Hence, it is unclear whether the differences in vitamin and minerals intakes between the IG and CG were exclusively due to whole grain consumption or the combined effect of having a healthier diet overall. Nonetheless, improved overall quality of diet has also been observed in similar studies of the differences between French [39] and Irish children [40] who were whole grain and non-whole grain consumers, where whole grain consumers showed significantly higher daily intakes of fiber and several minerals and vitamins compared with non-whole grain consumers. 

Although the present trial showed significant results on whole grain intake and dietary quality improvement in 12 weeks, the effects were diminished in the following six months. This indicates that adherence to a diet which emphasizes whole grain is weak post intervention. The initial higher levels of whole grain intake at T1 may be due to the novelty effect of consuming free whole grain food which was supplied and the fortnightly education classes. In fact, an earlier intervention aimed at improving the consumption of fruits among schoolchildren showed a result similar to our T1 results not being sustained at T2 [41]. It should be noted that whole grain foods are not only less available but also more expensive in Malaysia [30]. Whole grain foods availability and accessibility in the marketplace hinges on consumer demand along with cost, as well as the industry’s ability to produce whole grain foods and to maintain sufficient return on investment. Economic incentive policies to incorporate whole grain foods may be required to encourage higher availability of whole grain foods at a cheaper price. We opine that the Malaysian government should consider including whole grain food in its school feeding program, *Rancangan Makanan Tambahan*, for primary school children. In addition, public health strategies such as those used in Singapore, where vendors are subsided for offering whole grain food, should be also implemented in Malaysia. Although T1 results did not have sustainable effects at T2, the GReat-Child Trial helped in improving the overall whole grain intake and quality of diet among the children. Additionally, a follow-up of the GReat-Child Trial is in progress to investigate the sustainability of the trial [42].

Children increasingly consume and select their own favorite foods [13]. Hence, the quantity and quality of foods children consume should be evaluated through self-reports, as their caregivers may be unable to provide sufficient information on the children’s food intake. Self-reported dietary data include important information and have been used in numerous nutrition-related research works. However, errors always exist in such data [43]. Overweight or obese children have constantly reported similar or even less energy intake than their normal weight counterparts. It is unclear whether this situation is attributable to errors in the dietary assessment or such other reasons as day-to-day variations in food consumption, reporting to match social norms or difficulties in estimating portion size [43]. In spite of the challenges with dietary assessment, self-reported food intake remains a vital component of nutrition-related research [44,45]. To increase the accuracy of data from self-reported dietary intake, we excluded data indicating under- or over-reporting of energy intake from the data analysis.

Although the GReat-Child Trial findings are promising, there are numerous limitations. First, dietary assessment of children is challenging, as children, especially overweight and obese children, tend not to report their exact dietary intake due to their wish to adhere to social norms. In response to this limitation, we assured the participants of the confidentially of individual results and their anonymity. Secondly, a non-blinded technique, as opposed to a blinded technique, may over-estimate the effects of the trial. In circumstances when the participants are allocated to intervention, which involves dietary, behavioral, or educational change, it is not possible to blind the participants [46]. To diminish the limitation, we blinded the outcome assessors and statistician who conducted our data analyses. 

Despite all the above-mentioned limitations, the GReat-Child trial has several strengths. The trial represents a new approach to emphasize whole grain consumption in improving the quality of the diets of overweight and obese children. Outcomes of the present trial were adjusted by household income, a covariate which is likely to influence whole grain intake among Malaysian children [30].

## 5. Conclusions

The GReat-Child Trial showed that incorporating whole grain in a healthy balanced diet can significantly improve overall dietary quality in children who are overweight/obese. Moreover, outcomes from the GReat-Child trial showed that whole grain foods can be effectively included at home and in school. Greater accessibility to whole grain foods will result in improved whole grain intake by children. Changes brought about by the GReat-Child Trial are likely to improve the overall eating habits and subsequently the long-term health of children. The positive outcomes of the present trial have direct relevance to the school feeding program in Malaysia, which provides free meals to primary school students from low-income households. Future collaborative research endeavors among academia, industry, and government may result in the development of consumer- and cost-friendly whole-grain foods. We hope that the results from the present trial can be promoted at the national level and implemented in all schools by the Ministry of Education Malaysia. Doing so will increase the whole grain intake and reduce childhood obesity in Malaysian children. 

## Figures and Tables

**Table 1:** Baseline socio-demographics, physical characteristics, under- and over-reporting of energy intake and dietary intakes among children who successfully completed the entire trial (*n* = 63).

	Total (*n* = 63)	Intervention (*n* = 31)	Control (*n* = 32)	*p-*Value
Age; mean ± SD	10.6 ± 0.6	10.7 ± 0.6	10.6 ± 0.6	0.882 ^†^
Sex				0.262 ^††^
Boys; n (%)	33 (52.4)	18 (58.1)	15 (46.9)	
Girls; n (%)	30 (47.6)	13 (41.9)	17 (53.1)	
Household income; mean ± SD	4052 ± 1874	4507 ± 2385	3613 ± 1055	0.058 ^†^
Below MYR2300; n (%)	6 (9.5)	3(9.7)	3 (9.4)	0.452 ^††^
MYR2300-MYR5599; n (%)	50 (79.4)	23 (74.2)	27 (84.4)	
MYR5600 and above; n (%)	7 (11.1)	5 (16.1)	2 (6.2)	
Physical characteristics				
Weight (kg); mean ± SD	47.8 ± 13.0	50.4 ± 14.9	45.2 ± 10.4	0.486 ^†^
Height (cm); mean ± SD	139.8 ± 7.5	142.1 ± 8.2	137.5 ± 6.2	0.093 ^†^
BMI-for-age z-score; mean ± SD	2.2 ± 0.9	2.3 ± 1.0	2.1 ± 0.8	0.324 ^†^
Classification of energy-misreporting				0.368 ^††^
Under-reporting; n (%)	2 (3.2)	1 (3.2)	1 (3.1)	
Normal reporting; n (%)	59 (93.7)	30 (96.8)	29 (90.6)	
Over-reporting; n (%)	2(3.1)	0	2 (6.3)	
Dietary intake				
Energy (kcal); mean ± SD	2561 ± 367	2606 ± 363	2514 ± 370	0.511 ^†^
Protein (g); mean ± SD	96.1 ± 19.3	98.1 ± 23.7	94.1 ± 13.6	0.514 ^†^
Carbohydrate (g); mean ± SD	344.7 ± 56.1	352.5 ± 47.0	336.5 ± 64.1	0.428 ^†^
Fat (g); mean ± SD	88.6 ± 18.9	89.1 ± 22.8	88.1 ± 13.7	0.911 ^†^
Dietary fiber (g); mean ± SD	3.7 ± 2.7	3.9 ± 3.3	3.5 ± 1.9	0.386 ^†^
Thiamin (mg); mean ± SD	0.8 ± 0.2	0.8 ± 0.2	0.8 ± 0.2	0.325 ^†^
Riboflavin (mg); mean ± SD	1.0 ± 0.3	1.0 ± 0.2	1.1 ± 0.3	0.191 ^†^
Niacin (mg); mean ± SD	15.1 ± 4.5	15.4 ± 5.0	14.9 ± 4.0	0.940 ^†^
Calcium (mg); mean ± SD	402.2 ± 156.2	359.9 ± 128.8	446.0 ± 171.6	0.022 ^†,^*
Iron (mg); mean ± SD	31.7 ± 15.6	30.2 ± 15.4	33.4 ± 15.9	0.306 ^†^

#USD 1 = MYR 4.180 (as of 22 August 2020); ^†^ Tested using Chi-square test; ^††^ Tested using Independent *t*-test; SD: standard deviation; * *p*-value < 0.05; All the dietary intakes only involved 59 children (30 children from the intervention group; 29 children from the control group).

**Table 2:** Changes in whole grain and nutrient intakes within group (*n* = 59).

		Intervention Group		Control Group	
	Comparison	Mean (95% CI)	*p*-Value	Mean (95% CI)	*p*-Value
Whole grain (g)	T1-T0	20.6 (13.4, 27.9)	<0.001 ***	0	-
	T2-T0	8.8 (4.2, 13.5)	<0.001 ***	0	-
	T2-T1	−11.8 (−18.4, −5.3)	<0.001 ***	0	-
Energy (kcal)	T1-T0	−63(−182, 55)	0.553	96 (−31, 223)	0.192
	T2-T0	−41 (−136, 55)	0.851	192 (72, 312)	0.001 **
	T2-T1	22 (−51, 96)	1.000	96 (−18, 211)	0.123
Protein (g)	T1-T0	−3.2 (−9.2, 2.7)	0.524	−1.3 (−8.4, 5.8)	1.000
	T2-T0	−12.6 (−20.2, −5.0)	0.001 **	−1.0 (−8.3, 6.4)	1.000
	T2-T1	−9.4 (−17.3, −1.5)	0.016 *	0.4 (−6.0, 6.7)	1.000
Carbohydrate (g)	T1-T0	−11.0 (−37.0, 15.0)	0.865	19.5 (−2.0, 41.0)	0.085
	T2-T0	26.9 (5.1, 48.7)	0.012 *	40.5 (20.9, 60.1)	<0.001 ***
	T2-T1	37.9 (11.7, 64.2)	0.003 **	21.0 (−3.3, 45.3)	0.108
Fat (g)	T1-T0	−0.3 (−8.6, 8.0)	1.000	2.4 (−6.4, 11.2)	1.000
	T2-T0	−3.2 (−10.7, 4.4)	0.879	3.1 (−3.7, 9.9)	0.749
	T2-T1	−2.9 (−12.3, 6.5)	1.000	0.7 (−7.6, 9.1)	1.000
Fiber (g)	T1-T0	10.7 (6.1, 15.4)	<0.001 ***	3.5 (1.0, 5.9)	0.004 **
	T2-T0	3.7 (1.3, 6.4)	0.002 **	2.6 (0.5, 4.7)	0.014 *
	T2-T1	−7.0 (−11.9, −2.1)	0.003 **	0.9 (−4.6, 2.8)	1.000
Thiamin (mg)	T1-T0	0.8 (0.6, 0.9)	<0.001 ***	0.2 (0.1, 0.4)	<0.001 ***
	T2-T0	0.6 (0.4, 0.8)	<0.001 ***	0.2 (0.1, 0.3)	<0.001 ***
	T2-T1	−0.2 (−0.5, 0.1)	0.138	0.1 (−0.2, 0.1)	1.000
Riboflavin (mg)	T1-T0	1.9 (1.4, 2.4)	<0.001 ***	0.4 (0.1, 0.7)	0.004 **
	T2-T0	1.7 (−0.1, 3.5)	0.056	0.4 (0.1, 0.6)	0.003 **
	T2-T1	−0.2 (−2.1, 1.8)	1.000	−0.1 (−0.4, 0.3)	1.000
Niacin (mg)	T1-T0	9.8 (7.6, 12.0)	<0.001 ***	2.7 (0.5, 4.8)	0.010 *
	T2-T0	5.3 (0.4, 10.2)	0.029 *	1.1 (−0.5, 2.6)	0.255
	T2-T1	0.3 (−0.6, −0.1)	0.014 *	−0.1 (−0.4, 0.2)	0.679
Calcium (g)	T1-T0	404.0 (268.4, 539.7)	<0.001 ***	23.1 (−66.1, 112.2)	1.000
	T2-T0	199.5 (82.6, 316.4)	0.001 **	2.5 (−55.1, 60.1)	1.000
	T2-T1	−204.6 (−394.1, −15.0)	0.031 *	−20.6 (−128.3, 87.1)	1.000
Iron (g)	T1-T0	8.1 (1.0, 15.1)	1.000	−0.8 (−6.8, 5.2)	1.000
	T2-T0	9.7 (1.3, 18.2)	0.020 *	−0.1 (−5.6, 5.3)	1.000
	T2-T1	1.7 (−9.1, 12.5)	1.000	0.7 (−6.7, 8.0)	1.000

T0—Baseline; T1—post-intervention (thirteenth week); T2—follow-up (ninth month); * statistically significant at *p*-value < 0.05; ** statistically significant at *p*-value < 0.01; *** statistically significant at *p-*value < 0.001; Repeated measures ANCOVA within group analysis was applied followed by pairwise comparison with confidence interval adjustment; Household income and baseline variables as covariate.

**Table 3:** Comparison of whole grain and nutrient intakes over nine months between intervention and control groups (*n* = 59).

	Intervention Group—Control Group		
	Mean (95% CI)	*p*-Value	F-Stat(df)
Whole grain (g)	9.9 (7.1, 12.8)	<0.001 ***	50.19(1)
Energy (kcal)	−126 (−121, −40)	0.005 **	8.69(1)
Protein (g)	−3.4 (−8.1, 1.3)	0.156	2.07(1)
Carbohydrate (g)	−12.3 (−27.1, 2.5)	0.102	2.77(1)
Fat (g)	−5.3 (−10.6, 0.1)	0.055	3.85(1)
Fiber (g)	3.1 (1.4, 4.7)	0.001 **	13.60(1)
Thiamin (mg)	0.3 (0.2, 0.4)	<0.001 ***	39.51(1)
Riboflavin (mg)	0.8 (0.4, 1.3)	0.001 **	12.71(1)
Niacin (mg)	0.4 (1.9, 5.2)	<0.001 ***	19.01(1)
Calcium (mg)	130.3 (74.2, 186.4)	<0.001 ***	21.64(1)
Iron (mg)	2.2 (−1.7, 6.1)	0.258	1.31(1)

Repeated measures ANCOVA between group analyses was applied followed by pairwise comparison; Household income and baseline variables as covariate; ** statistically significant at *p*-value < 0.01; *** statistically significant at *p-*value < 0.001.
